# Leveraging Accelerometry as a Prognostic Indicator for Increase in Opioid Withdrawal Symptoms

**DOI:** 10.3390/bios12110924

**Published:** 2022-10-26

**Authors:** Tamara P. Lambert, Asim H. Gazi, Anna B. Harrison, Sevda Gharehbaghi, Michael Chan, Malik Obideen, Parvaneh Alavi, Nancy Murrah, Lucy Shallenberger, Emily G. Driggers, Rebeca Alvarado Ortega, Brianna Washington, Kevin M. Walton, Yi-Lang Tang, Rahul Gupta, Jonathon A. Nye, Justine W. Welsh, Viola Vaccarino, Amit J. Shah, J. Douglas Bremner, Omer T. Inan

**Affiliations:** 1 The Wallace H. Coulter Department of Biomedical Engineering, Georgia Institute of Technology, Atlanta, GA 30332, USA; 2 School of Electrical and Computer Engineering, Georgia Institute of Technology, Atlanta, GA 30332, USA; 3 Department of Psychiatry and Behavioral Sciences, Emory University School of Medicine, Atlanta, GA 30322, USA; 4 Department of Epidemiology, Rollins School of Public Health, Atlanta, GA 30322, USA; 5 Clinical Research Grants Branch, Division of Therapeutics and Medical Consequences, National Institute on Drug Abuse, Bethesda, MD 20877, USA; 6 Atlanta VA Medical Center, Decatur, GA 30033, USA; 7 Department of Radiology, Emory University School of Medicine, Atlanta, GA 30322, USA; 8 Division of Cardiology, Department of Internal Medicine, Emory University School of Medicine, Atlanta, GA 30322, USA

**Keywords:** opioid use disorder, opioid withdrawal, accelerometer, wearable, spectrograms, spectral analysis, opioid addiction, non-invasive therapies, vagal nerve stimulation, restlessness

## Abstract

Treating opioid use disorder (OUD) is a significant healthcare challenge in the United States. Remaining abstinent from opioids is challenging for individuals with OUD due to withdrawal symptoms that include restlessness. However, to our knowledge, studies of acute withdrawal have not quantified restlessness using involuntary movements. We hypothesized that wearable accelerometry placed mid-sternum could be used to detect withdrawal-related restlessness in patients with OUD. To study this, 23 patients with OUD undergoing active withdrawal participated in a protocol involving wearable accelerometry, opioid cues to elicit craving, and non-invasive Vagal Nerve Stimulation (nVNS) to dampen withdrawal symptoms. Using accelerometry signals, we analyzed how movements correlated with changes in acute withdrawal severity, measured by the Clinical Opioid Withdrawal Scale (COWS). Our results revealed that patients demonstrating sinusoidal–i.e., predominantly single-frequency oscillation patterns in their motion almost exclusively demonstrated an increase in the COWS, and a strong relationship between the maximum power spectral density and increased withdrawal over time, measured by the COWS (R = 0.92, *p* = 0.029). Accelerometry may be used in an ambulatory setting to indicate the increased intensity of a patient’s withdrawal symptoms, providing an objective, readily-measurable marker that may be captured ubiquitously.

## 1. Introduction

The opioid overdose epidemic is a major crisis in the United States, with a nearly five-fold increase in natural and semi-synthetic opioid overdose-related deaths over the past 20 years [1]. A mainstay of treatment is medications for Opioid Use Disorders (MOUD), including buprenorphine, methadone, and naltrexone [2,3,4,5,6]. Identified barriers to treatment include actual or fear of physical discomfort associated with acute opioid withdrawal, including sympathetic arousal, cardiovascular activation including increased heart rate and blood pressure, anxiety, nausea, and restlessness [2], in addition to limitations in attaining access to MOUD due to cost, limited treatment resources and access, and stigma [4]. Naltrexone may be preferable for patients with OUD who want to avoid treatment with opioid agonists and partial agonists such as methadone or buprenorphine [7,8]. Since naltrexone acts as an opioid receptor antagonist, however, administration can precipitate acute withdrawal if patients are continuing to use opioids. Initiation of naltrexone in someone with active opioid use therefore requires an extended window of opioid withdrawal, which represents a window of vulnerability to relapse and increased risk of overdose-related death due to a resetting of the opioid receptor [9,10].

Opioid withdrawal can be assessed by rating scales such as the COWS scale [11]. The COWS includes subjective withdrawal symptoms as well as ratings of opioid withdrawal severity by an external observer [11]. These ratings rely on subjective self-report and clinical observation which may be vulnerable to biases in assessment [12,13,14,15,16]. For these reasons, subjective ratings may not always accurately estimate the level of withdrawal that a patient is experiencing. Additionally, these surveys primarily focus on identifying the current withdrawal state of a patient, but do not indicate the potential of a patient’s symptoms worsening. Objective measures of withdrawal based on physiological measurements may not only be useful in the assessment and treatment of patients with OUD in acute opioid withdrawal but provide prognostic value for symptoms potentially worsening.

One of the elements of opioid withdrawal captured by rating scales such as the COWS is the restlessness that may be associated with involuntary movements. Although anecdotal clinical observations have noted an increase in these movements in patients with acute opioid withdrawal, studies have not previously assessed these movements in detail and quantitative information is lacking. Little is known about the mechanistic origins of these movements, however acute withdrawal of opioids in patients with OUD may lead to activation of μ-opioid receptors in the mesolimbic pathway, resulting in dopamine deficiencies and the manifestation of movement pathologies similar to what is seen in disorders such as Parkinson’s Disease (PD) and restless leg syndrome (RLS) [17]. Quantification of these types of involuntary movements can be achieved using sensing devices such as accelerometers.

Using wearable technology to monitor OUD is a recent innovation compared to subjective surveys, and several studies focus on the use of wearables to monitor physiological changes occurring during opioid intake [16,18,19,20]. Some studies have used quantitative physiological measures to assess objective manifestations of drug withdrawal. One study used accelerometry to show a decrease in psychomotor arm steadiness (arm-droop) and an increase in tremor in current and former recreational stimulant users versus those who have never used stimulants [21]. Other studies compared the bodily movements of abstinent stimulant users with nonuser controls [22,23], sleep disturbance in patients using opioids [24,25,26,27], or non-compliance with biosensor use in patients with OUD [28]. Other studies have used alternate methods to analyze opioid withdrawal, but these methods primarily focus on withdrawal in neonates, and may not be as feasible to use to monitor a patient as they go about their daily activities due to requiring video tracking and patient samples to analyze genetic expression [29,30,31].

Two studies involve the use of wearables to monitor the opioid withdrawal induced by naloxone, an opioid reversal antidote functioning by blocking the opioid receptors. The first study involved patients with OUD who received naloxone after an opioid overdose using an algorithm of physiological data including blood volume pulse, electrodermal activity, skin temperature, and body movement measured with an accelerometer as a predictor of presence or absence of opioid withdrawal [32]. The second study involved evaluating physiological data such as heart rate, temperature, electrodermal activity, and accelerometry extracted from wrist-mounted biosensors to determine the point at which naloxone loses its effect after administration to the patient [33]. However, the primary focus of the first study was on withdrawal detection, rather than elucidating relationships between changes in the biometric data and withdrawal over time in patients electively entering an opioid withdrawal condition after discontinuation of use, a more clinically relevant scenario for patients with OUD [32]. The second study primarily focused on determining physiological landmarks that mark the cessation of the effects of naloxone in patients who have overdosed on opiates rather than monitoring opioid withdrawal induced by discontinuation of use over several hours [33]. Given the limited research available regarding using wearables to study opioid withdrawal and the potential for far-reaching impact, there is a need for novel approaches to evaluate the role of wearable technology in combating OUD.

**Figure 1:**
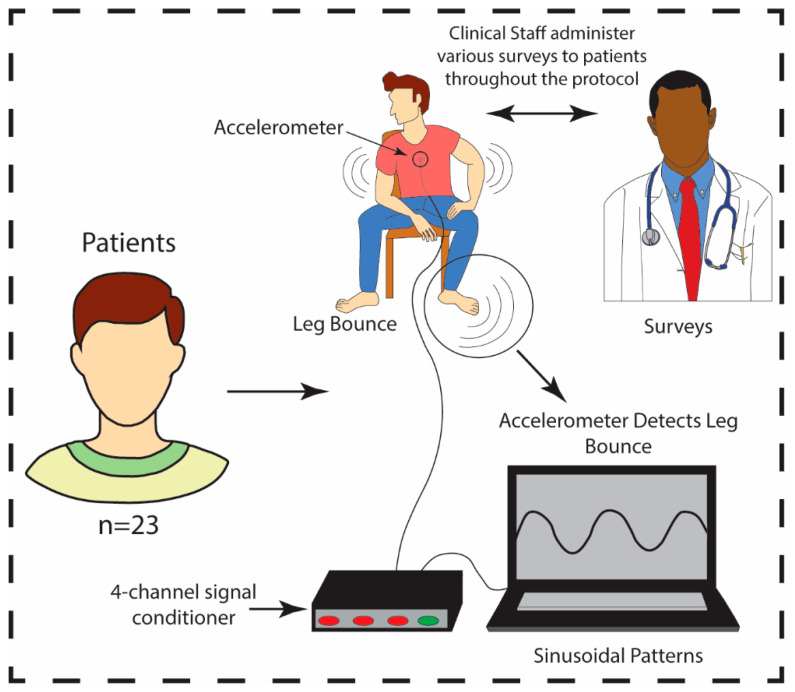
A sample size of *n* = 23 patients participated in the protocol. Patients were administered COWS by the clinical staff throughout the protocol while patient movements were tracked via accelerometry. Movement data was monitored by a triaxial accelerometer connected to a 4-channel signal conditioner powering the accelerometer. The entire unit was connected to a laptop. The accelerometer was positioned at the center of the sternum and recorded in real-time.

Accelerometry may provide a reliable, quantitative method of measuring involuntary movements as an objective marker of the severity of opioid withdrawal in patients experiencing acute opioid withdrawal. This methodology may offer innovation in assessment, treatment monitoring, and determination of prognosis in patients with OUD. This study used accelerometry to assess involuntary movements in patients with OUD in acute opioid withdrawal. We focused on periodic leg bouncing/foot tapping (LBFT) exhibited by a portion of the patient sample. These movements were displayed as sinusoidal patterns, demonstrated in Figure 1.

We focused on LBFT for analysis as this was a distinct pattern exemplified by a sub-group of subjects. LBFT is commonly associated with anxiety [34], which is one of the items used in COWS to evaluate levels of withdrawal. Other motion such as shifting was observed across a subset of subjects, but was typically necessitated by the protocol such as the need to re-position the sensors and leaning closer to the computer monitor to view the opioid cues. We hypothesized that patients experiencing symptoms of opioid withdrawal as measured by the COWS scores would also demonstrate increased strength and frequency of involuntary movements as measured by accelerometry. We examined patients with OUD during opioid withdrawal and exposure to opioid cues, and classified them as “Sinusoidal” or “Non-Sinusoidal” based on the presence or absence of sinusoidal patterns in their accelerometry data. Afterward, we extracted frequency and maximum power spectral density (max PSD) from select portions of patient data and conducted correlation analyses between frequency and max PSD, and the overall COWS scores and max PSD. Our aim was to determine the strength and significance of the correlation between the levels of withdrawal a patient is experiencing and the intensity of their movements, and if the intensity of their movements increases with the frequencies of their movements.

## 2. Materials and Methods

### 2.1. Patient Study Sample and Assessments

This was a double-blind sham-controlled study of transcutaneous Vagal Nerve Stimulation (tcVNS) for acute opioid withdrawal in patients with Opioid Use Disorder (OUD). This study was registered on ClinicalTrials.gov as NCT04556552 and was approved by the Institutional Review Boards of the Georgia Institute of Technology (H20203) and Emory University School of Medicine (IRB00117320). Patients experiencing acute opioid withdrawal underwent the same protocol as previously described [35]. Acute withdrawal is defined as being abstinent from opioid use for at least 8 h before protocol initiation. Patients met the criteria for OUD based on the Structured Clinical Interview for the Diagnostic and Statistical Manual of Mental Disorders, Fifth Edition (DSM-5) [5,36], and were between the ages of 18 and 80 years old. Patients were excluded if they had a history of the following conditions: schizophrenia, schizoaffective disorder, cervical vagotomy, traumatic brain injury, meningitis, loss of consciousness for greater than one minute, bulimia, neurological disorder, serious medical or neurological illness, carotid atherosclerosis, currently pregnant, breastfeeding or were implanted with a device (e.g., pacemaker). The COWS survey is an 11-item survey administered by a clinician at the beginning and end of the protocol to assess the severity of OUD experienced by the patient. The items are rated on a scale from 0–4 (or 0–5 dependent on the question). The total score ranges from 0 to 48, with 0 indicative of no opioid withdrawal symptoms, and 48 indicative of the most severe opioid withdrawal symptoms.

### 2.2. Creating Spectrograms from the Accelerometer Data

**Figure 2:**
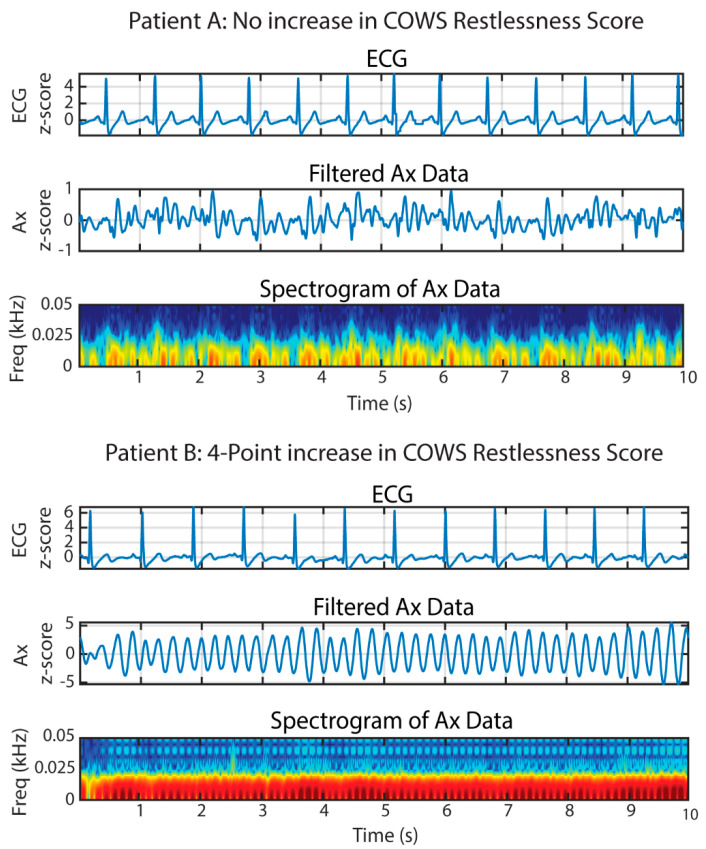
Spectral analysis of accelerometer waveforms extracted from 10−s windows during the experiment. Patient A represents patients not exhibiting sinusoidal wave patterns and having no increase in their COWS restlessness scores, while Patient B represents patients exhibiting sinusoidal wave patterns and having a four-point increase in their COWS scores. The amplitudes of the electrocardiogram (ECG) and accelerometer signals in the Ax-direction were normalized using z-score normalization as indicated by the y-axes in the figure. All accelerometer waveforms are taken from the craniocaudal direction. The dark red color where the highest energy content is located (and where LBFT is located) is between 0−10 Hz. The other colors such as yellow and blue are low energy noise signals.

The analysis focused specifically on the low-noise 356A32 3-axis accelerometer (PCB Piezotronics, Depew, NY, USA) data collected at the center of the sternum (placement shown in Figure 1), placed under the shirt in contact with the skin (placement shown in Figure 1). The accelerometer had a sensitivity of 100 mV/g ± 10% and a measurement range of ±50 g. This accelerometer was chosen due to the need to capture sub-Hz level vibrations that are required when sensing cardiac vibrations relevant to exploratory endpoints for our previous study [35]. As the posture of the subject did not change dynamically in our study, solely measuring dynamic accelerations was sufficient. Data were collected at a sampling frequency (fs) of 2 kHz using the Biopac MP150 data acquisition system. Signal processing and data analysis were conducted using MATLAB (R2021a, Natick, MA, USA) and Microsoft 365 (Excel, Version 2110, Redmond, WA, USA). Accelerometer signals were extracted from the craniocaudal (Ax-direction) as we hypothesized the majority of the power of the movement would be found in the Ax-direction due to the nature of the movement. The alignment of the accelerometer was maintained using markers indicative of the direction on the accelerometer. To create the spectrograms for the accelerometer data (Figure 2), Ax was filtered in MATLAB using a 3rd order Butterworth filter and the following parameter: lowpass filter cutoff (fL) = 50 Hz. This cutoff was chosen during our initial exploratory analysis for visualization purposes and to ensure that there was no significant frequency content occurring above 10 Hz. The data were then filtered using zero-phase digital filtering to receive the final filtered version of the accelerometer data in the Ax-direction. The accelerometer and electrocardiogram data (ECG) data were subsequently normalized using z-score normalization. Afterward, the ECG data, filtered accelerometer data, and the spectrogram of the accelerometer data were plotted simultaneously using 10-s windows, and the plots were examined for the presence of sinusoidal patterns.

**Figure 3:**
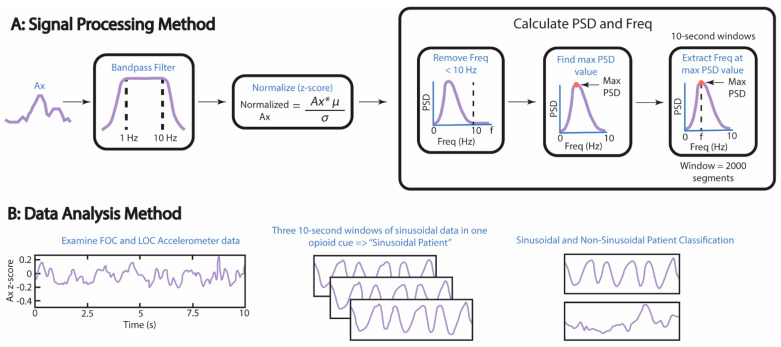
Methods used to find significant correlations between the COWS scores and the normalized max PSD, as well as the correlations between the frequency at the max PSD and the normalized max PSD. (**A**) Accelerometer data were normalized using z-score normalization, where µ = mean and σ = standard deviation. The maximum PSD of a 10−s window was normalized to the summation of all PSD values within the same window. Freq = frequency. (**B**) Processed data were analyzed by visual inspection for sinusoidal patterns in the accelerometer data lasting ≥5 s in a 10−s window. A patient was labeled a “Sinusoidal Patient” if three or more windows containing the aforementioned pattern were found in the first opioid cue (FOC) or last opioid cue (LOC) of their accelerometer data.

The first and last opioid cues, videos containing images of opioid usage, were selected for examination to compare the difference in patient response to opioid cues over time. Each patient was examined for the presence of sinusoidal waveforms in their accelerometer data via visual inspection, comparing the accelerometer waveforms to the ECG waveforms to verify that the accelerometer waveform was not a result of cardiac muscle contraction. Data was collected from a total of 23 patients. Two patients were excluded from examination due to equipment malfunctions resulting in the loss of timing information and corruption of accelerometer data, respectively, resulting in 21 patients being included in data analysis. The average age of the patients was 35.4 ± 10.7 years. Additional demographic and study details are included in Table S1 (Supplementary Materials). Patients’ data were segmented into 10-s windows for inspection. Each 10-s window that contained longer than 5 s of continuous sinusoidal data were marked as “sinusoidal” (refer to Figure 3B for examples of sinusoidal patterns). If a patient had three or more “sinusoidal” windows in the FOC or the LOC, the patient was labeled “Sinusoidal.” Three 10-s timeframes per opioid cue were chosen based on which timeframes exhibited the most sinusoidal patterns visually. If the patient displayed two or fewer 10-s windows containing the aforementioned specifications for sinusoidal patterns in the FOC and the LOC, the patient was classified as “Non-Sinusoidal.” For “Non-Sinusoidal” patients, the specific timeframes for three 10-s windows containing non-sinusoidal data were extracted per opioid cue.

### 2.3. Processing the Accelerometer Data to Extract Frequency and Maximum Power Spectral Density

To process the accelerometer data as described in Figure 3A, Ax was bandpass filtered using the following parameters: lower frequency limit of the bandpass filter (f_L_) = 1 Hz, upper frequency limit of the bandpass filter (f_U_) = 10 Hz, and infinite impulse response. A bandwidth of 1 to 10 Hz was chosen because the frequency of LBFT was expected to be between 1 and 10 Hz. The ECG and the filtered Ax signal were normalized using z-score normalization. Welch’s power spectral density estimate (pwelch) was used to calculate the power spectral density (PSD) and the frequency at the max PSD (F). The following parameters were used to calculate PSD and F: filtered Ax data, window = 2000 segments, number of overlapped samples = 500 samples, and number of discrete Fourier transform points = 10,000. The filtered Ax data was indexed to calculate the PSD, and the mean of the indexed, filtered PSD was subtracted to control for DC offset. All frequencies equal to or greater than 10 Hz were removed, as well as PSDs located at frequencies equal to or greater than 10 Hz. The maximum PSD was then calculated per window, and data were normalized by dividing the max PSD per window by the summation of all of the PSD values in the filtered Ax data at frequencies <10 Hz. Normalization was performed to make the max PSD values comparable across all windows and to determine the sinusoidal regularity of the signal. The ECG data, filtered accelerometer data, and the normalized max PSD of the accelerometer data was plotted using the same technique mentioned in the previous paragraph for visualization purposes, with the exception of plotting the PSD on a log scale.

### 2.4. Analysis Methods of the Accelerometer Data

To analyze the accelerometer data as described in Figure 3B, the three normalized max PSD data points extracted from each opioid cue were averaged per opioid cue. This was done to create one data point per opioid cue for each patient. These data points were graphed against the change in COWS scores. The change in COWS scores is defined as the difference between the COWS scores taken at the beginning and the end of the experimental protocol. Additionally, the frequencies at the max PSD within the three selected windows were extracted and then graphed against the max PSD. Correlation coefficients and *p*-values were calculated using Pearson’s R as the sample satisfied several parameters, including normality of residuals using the Shapiro–Wilk test to determine the statistical significance (*p*-value = 0.05). Homoscedasticity was more difficult to determine due to the limited amount of data points available, but homoscedasticity was assumed for each plot after examination of the scatter plots for each opioid cue and patient classification.

## 3. Results

### 3.1. Classification of Patients as “Sinusoidal” vs. “Non-Sinusoidal”

**Figure 4:**
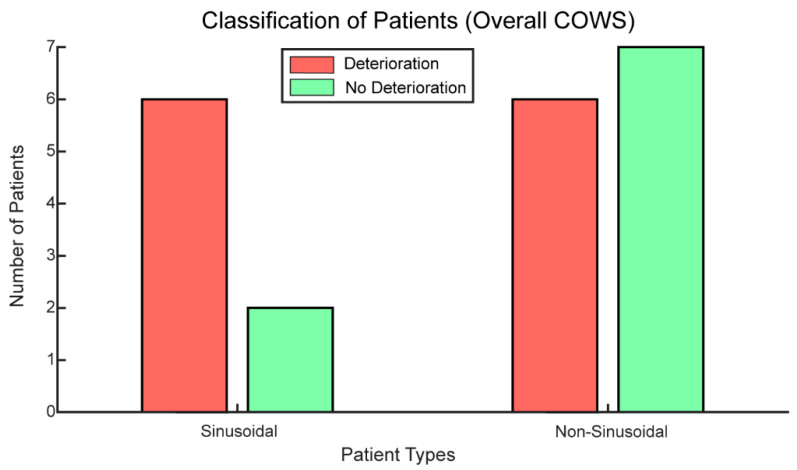
Most patients with OUD classified as “Sinusoidal” had an increase in opioid withdrawal or deterioration over time as measured with the COWS. Patients classified as “Non-Sinusoidal” were as likely to have worsening withdrawal symptoms (deterioration) as no change or an improvement in withdrawal symptoms (no deterioration: no change or a decrease in their overall COWS scores).

Seventy-five percent of patients exhibiting sinusoidal patterns in their accelerometer signals demonstrated an increase in overall COWS scores. As demonstrated in Figure 4, most of the patients with OUD classified as “Sinusoidal” exhibited an increase in withdrawal over time as measured with the COWS score. Patients classified as non-sinusoidal were about half as likely to experience an increase in their COWS score as to not experience an increase in their COWS score (six versus seven respectively).

### 3.2. Significant Correlations Are Typically Found in the LOC

When examining the correlation between overall COWS score change pre and post protocol, and normalized max PSD, the criteria for inclusion were: (1) patients had at least three 10-s windows that contained longer than 5 s of continuous sinusoidal data, and (2) patients had a positive COWS score indicative of deterioration in their withdrawal state. Therefore, for the FOC and the LOC, the number of data points evaluated equals five (*n* = 5). When comparing the overall COWS score changes to the normalized max PSD, we saw that there was no significant relationship between the two variables for the FOC (R = 0.20, *p*-value = 0.74), but there was a significant relationship for the LOC (R = 0.92, *p*-value = 0.029) (Table 1). This indicates that the power, or strength of the foot tapping increases as the overall COWS score change increases, or as the patient experiences deterioration in their withdrawal state. These results also suggest that this relationship occurs temporally. Towards the end of the protocol, the relationship between the increase in COWS scores and normalized max PSD strengthens significantly. This may indicate that accelerometry becomes more prognostically indicative of an increase in COWS scores as time progresses.

When examining the correlation between frequency and normalized max PSD, the criteria for inclusion in the FOC was (1) patients had at least three 10-s windows that contained longer than 5 s of continuous sinusoidal data. The LOC had the additional inclusion criteria that (2) patients had a positive COWS score indicative of deterioration in their withdrawal state. Therefore, for the FOC *n* = 7 and for the LOC, *n* = 5. When comparing frequency to normalized max PSD for the LOC, we found that there was a statistically insignificant relationship between frequency and max PSD when including patients that had a negative difference between their pre- and post-COWS scores (R = 0.69, *p*-value = 0.085). However, when we excluded patients with a negative overall change in COWS scores, we have a very strong and significant relationship between frequency and max PSD (R = 0.96, *p*-value = 0.0086) (Table 2).

This indicates that as patients with opioid withdrawal increase the rate of LBFT, the strength behind the foot tapping increases. These results also suggest that the frequency increasing with normalized max PSD is dependent on the patient experiencing a deterioration in their withdrawal state. This result stratifies for an increase in COWS scores (deterioration) versus all COWS scores. As only two individuals with sinusoidal waveforms had negative COWS scores (decreased withdrawal), they were not analyzed separately.

### 3.3. Demographic Prognostic Indicators of Sinusoidal and Non-Sinusoidal Accelerometer Waveforms

Patients displaying sinusoidal waveforms in the FOC and the LOC tended to almost exclusively be under 35 years old, unmarried, and had a comorbid depressive disorder. Out of the eight patients that were classified as “Sinusoidal”, only one patient was over the age of 35. Comparing the patients classified as “Non-Sinusoidal,” about half were under the age of 35 as were over the age of 35 (seven versus six patients respectively). When considering marital status, almost all of the patients displaying sinusoidal waveforms in their accelerometer data were not married (seven versus one patient respectively) while those displaying non-sinusoidal waveforms were about twice as likely to not be married. For the patients that have recorded Structured Clinical Interview for DSM-5 (SCID-5) data, all seven “sinusoidal patients” reported a depressive disorder, and four out of eight “non-sinusoidal patients” reported a depressive disorder. Other demographic features (race, gender, education, employment) did not yield notable results.

## 4. Discussion

Our results suggest that sinusoidal waveforms in accelerometer data may be prognostically indicative of an increase in opioid withdrawal severity as measured with the COWS in patients with OUD (Figure 4). There are several possible explanations for why this would occur. One is that those who demonstrated sinusoidal waveforms in their accelerometer data were more likely to have a past or current depressive disorder documented in their SCID-5. A positive and statistically significant association has been demonstrated between depression and the increased prevalence of restless leg syndrome (RLS) [37,38], which may shed some light on the LBFT demonstrated by “sinusoidal patients.” The compounding effects of depression in addition to the lack of dopamine release due to opioid withdrawal may be inducing an RLS-like syndrome in patients demonstrating LBFT.

Another possible explanation for our findings may be related to the neurobiology of opioid withdrawal. The sinusoidal movement patterns have a similar periodic waveform to tremor, which is a condition that can develop due to dopamine deficiency [39,40]. Substances such as cocaine and heroin may lead to movement disorders in the form of tremor or Parkinsonism [41]. Cocaine and heroin use may both lead to dopamine deficiencies [6]. While cocaine may induce dopamine deficiency due to blockage of the dopamine transporter, preventing dopamine reuptake, opioids such as heroin bind to the μ-opioid receptor causing dopamine to flood the system and eventually leading to downregulation of dopamine receptors with prolonged usage [41,42]. Higher percentages of “Sinusoidal Patients” reported usage of both drugs compared to “Non-Sinusoidal Patients” (62.5% vs. 23.1% respectively), which may potentially indicate synergistic interactions between the drugs that present as LBFT. Other potential mental disorders such as ADHD were considered as a possible explanation for the LBFT, but further examination of the SCID-5 did not reveal any additional shared mental disorders.

Additionally, it is important to note that time seemed to be an influential component in the significance of the relationship between the COWS scores increase and the max PSD, as well as the relationship between the max PSD and the frequency of the movements. Opioid withdrawal typically follows a predictable time course, mostly based on the half-life of the opioid used. Withdrawal for short-acting opioids typically takes place 8–24 h post final use and often lasts for 4–10 days, while withdrawal for long-acting opioids takes place 12–48 h post final use and can last up to 10–20 days [43]. The patients recruited were ≥8 h post last use of opioids, and therefore withdrawal may not have completely set in during the FOC, which was closer to the beginning of the protocol (64-min mark). The LOC occurs around the 90-min mark, just before the end of the protocol. Therefore, it is possible that the patients are experiencing more discomfort due to opioid withdrawal as time progresses, which may present as stronger and more frequent LBFT movements in the accelerometer data and increases in the strength of LBFT movements as the COWS score increases.

There are several limitations to our study. Our study has a relatively small sample size (*n* = 23), and therefore there might be a lack of generalizability of our findings to a larger population. Future studies would need to include more patients. Another limitation present in our study is that our correlation analyses have a small sample size. This also may limit the generalizability of our conclusions, and therefore a larger sample of OUD patients that demonstrate sinusoidal wave patterns in their accelerometer results and deterioration in their opioid withdrawal state would need to be included to verify our results. However, as our analysis includes multiple timeframes of sinusoidal patterns per subject to ensure the robustness of our results, our analysis serves as a starting point for future studies to validate our findings. Comparing those who experienced an increase in opioid withdrawal symptoms, patients were half as likely to display sinusoidal patterns as they were to not display sinusoidal patterns. While examining sinusoidal movement may only identify half of the patients with worsening withdrawal, this rapid analysis could relieve some of the clinical burden of identifying those who may need additional care. Another limitation is that our analysis was restricted to examining accelerometer data in the FOC and the LOC, thus it is possible that there were undetected sinusoidal patterns in the stimuli in between the FOC and LOC and therefore may have changed the nature of our analysis. However, because our main focus was on understanding how the patient’s accelerometer data changed as a result of exposure to nVNS at the beginning and the end of the protocol, this data analysis was excluded.

The reported max PSD values and their corresponding frequencies for each patient were computed by averaging three select 10-s windows determined by the criteria outlined in the Materials and Methods section. There is potential that the max PSD and frequency values may change if more windows are included and therefore, additional windows may need to be included in future analyses for more accurate estimates of these values. Moreover, statistical testing has determined that there is no statistically significant difference in the levels of deterioration between patients who displayed sinusoidal patterns versus those who did not. However, this result may be due to the small sample size, and additional data points may confirm our result at a statistically significant level. This also may indicate that sinusoidal pattern may potentially be a contributor to a larger, multivariate algorithm that can accurately determine a patient with OUD’s probability of deterioration. The alignment of our accelerometer may be another limitation due to human error in placement. However, we have mitigated for this potential source of error through directionality markings on our accelerometer and ensuring the same placement for each subject. Future studies may include the evaluation of how sensor placement impacts the results. Another limitation for consideration is the accelerometer chosen for our study was not optimized for movement detection as it did not include DC acceleration (i.e., to enable posture detection). A further limitation of our study is the lack of specificity of anxiety and restlessness to opioids as these symptoms may be present due to withdrawal from other substances [44,45]. Moving forward, this limitation may be addressed by assessing the presence of sinusoidal patterns in patients by including patients who only use one type of substance, and stratifying the results based on the type of substance used.

## 5. Conclusions

Our study demonstrates that accelerometry may be used as an objective marker of opioid withdrawal in patients with OUD. Accelerometry has demonstrated a wide variety of human health applications, including substance use and its behavioral impact [16,18,19,20,21,22,23,24,25,26,27,28], and stress, which is a common symptom of a variety of environmental exposures including opioid withdrawal [46,47,48,49]. Nevertheless, few studies have used accelerometry to study opioid withdrawal [32,33]. To our knowledge, the link between OUD and LBFT, a symptom that may be induced by opioid withdrawal has yet to be explored using accelerometry [17]. We show that LBFT may potentially indicate the worsening of withdrawal symptoms, with most patients classified as “Sinusoidal” demonstrating an increase in their COWS (6 out of 8 patients). Our results also demonstrate that LBFT detected by accelerometry may have a positive, statistically significant relationship with elevated COWS scores temporally (R = 0.92, *p* = 0.03 for LOC). These results indicate the potential to use accelerometry to determine if a patient is at risk of worsening withdrawal and may require additional symptom management. Our findings also demonstrate a relationship between the presence of sinusoidal waveforms in the accelerometer data and age, marital status, and co-morbid depressive disorders.

Our work serves as a starting point for ways in which accelerometry may be used as a prognostic tool to identify patients who may experience an increase in opioid withdrawal. The results we received from our analysis of LBFT using accelerometry demonstrate the potential to be combined with other potential risk factors, and used to identify patients at increased risk of deterioration and relapse. Our results demonstrate the need for further research in the use of wearables to monitor opioid withdrawal, and may lead to the development of non-invasive, real-time monitoring systems for measuring and predicting deterioration in opioid withdrawal levels in the future. This research may contribute to the development of more objective measures of opioid withdrawal, which is currently heavily dependent on subjective measurement scales and prone to bias [12,13,14,15,16]. Additionally, this information may serve as an early warning signal to clinicians of the necessity to provide higher-risk patients with additional care to optimize treatment and prevent relapse with the potential for overdose.

## Figures and Tables

**Table 1:** A significant correlation between the overall COWS scores changes and the normalized max PSD occurred during the LOC for “Sinusoidal Patients” with positive COWS scores, indicating deterioration of “Sinusoidal Patients” with positive COWS scores temporally.

	N	Δ COWS Scores *^a^*	Normalized Max PSD *^b^*	Pearson’s R	*p*-Value
FOC *^c^*	5	5.40 ± 3.29	0.11 ± 0.02	0.20	0.74
LOC *^d^*	5	5.60 ± 3.05	0.11 ± 0.02	0.92	0.03

*^a^* Δ COWS Scores = change in clinical opiate withdrawal scale; *^b^* Normalized Max PSD = normalized maximum power spectral density; *^c^* FOC = first opioid cue; *^d^* LOC = last opioid cue.

**Table 2:** “Sinusoidal Patients” experiencing deterioration experienced a significant increase in the frequency of their motion as the power in their motion increased. Det. = deterioration.

	N	Frequency (Hz)	Normalized Max PSD	Pearson’s R	*p*-Value
All Patients	7	4.48 ± 1.37	0.11 ± 0.02	0.69	0.09
Det. Only	5	3.39 ± 1.12	0.11 ± 0.02	0.96	0.009

## Data Availability

Data are available on request due to privacy restrictions.

## Supplementary Materials

The following supporting information can be downloaded at: https://www.mdpi.com/article/10.3390/bios12110924/s1, Table S1: Patient Demographics and Study Details.
