# Supplementary material for: Leveraging Accelerometry as a Prognostic Indicator for Increase in Opioid Withdrawal Symptoms
Source: Biosensors (Basel). 2022 Oct 26;12(11):924. doi: 10.3390/bios12110924 (PMC9688173; doi:10.3390/bios12110924)
Supplement: Supplementary file 1 [file biosensors-12-00924-s001.zip › biosensors-1917095-supplementary.pdf]

Leveraging Accelerometry as a Prognostic Indicator for Increase in Opioid Withdrawal Symptoms

Supplementary Material

Table S1. Patient Demographics and Study Details

Participant Number	Age [y]	Sex [F/M]	Race and Ethnicity	Education [y]	Employment Status	Marital Status	Drugs Used	ΔCOWS Scores	Sinusoidal or Non-Sinusoidal	MHD[a] (Excluding D/A[b] Use)	Study Location
1	27	F	White	14	Full Time	Never Married	oxycodone, heroin	3	Sinusoidal	DD[c], PTSD, ADHD	Emory
2	52	M	Black	14	Full Time	Married	Oxycodone	-2	Non-Sinusoidal	N/A*[d]	Emory
3	37	M	White	12	Full Time	Married	DNR	-2	Non-Sinusoidal	DNR[e]	Emory
4	35	M	Hispanic	12	Full Time	Never Married	Cannabis, heroin	8	Sinusoidal	DD	Alliance
5	34	F	Black	12	Unemployed	Never Married	Oxycodone	3	Sinusoidal	DD	Emory
6	52	M	White	8	Unemployed	Never Married	Heroin, cocaine	7	Non-Sinusoidal	N/A*	Emory
7	31	F	White	12	Unemployed	Married	Marijuana, Adderall, methamphetamine, oxycodone	-4	Non-Sinusoidal	DD	Emory
8	27	F	Asian	12	Unemployed	Never Married	Marijuana, oxycodone	6	Sinusoidal	DD	Emory
9	37	F	Black	12	Disabled	Divorced	Oxycodone	-6	Non-Sinusoidal	DD, PTSD	Emory
10	24	M	Asian	12	Full Time	Never Married	Sedatives, Marijuana, Oxycodone, Mushroom, Kratom	0	Non-Sinusoidal	DD	Emory
11	32	M	Hispanic	12	Part Time	Never Married	Heroin, Marijuana, Amphetamine, Adderall, Methamphetamine, Cocaine, Sedatives	-1	Sinusoidal	DD	Emory

12	23	M	Black	12	Part Time	Never Married	Marijuana, Amphetamine, Adderall, Oxycodone	2	Sinusoidal	DD, ADHD	Alliance
13	29	M	Black	12	N/A	Never Married	DNR	1	Non-Sinusoidal	DNR	Alliance
14	29	M	Black	12	Full Time	Never Married	DNR	-1	Non-Sinusoidal	DNR	Alliance
15	28	M	White	12	Unemployed	Never Married	DNR	2	Sinusoidal	DNR	Alliance
16	27	F	Hispanic	12	Student	Never Married	DNR	1	Non-Sinusoidal	DNR	Alliance
17	27	M	White	N/A	N/A	Never Married	DNR	9	Non-Sinusoidal	DNR	Alliance
18	47	M	White	12	Part Time	Married	DNR	1	Non-Sinusoidal	DNR	Emory
19	59	M	White	14	Disabled	Never Married	DNR	0	Non-Sinusoidal	DNR	Alliance
20	52	M	Black	12	Disabled	Married	Sedatives, marijuana, heroin, Xanax	-1	Sinusoidal	DD, PD[g]	Alliance
21	34	M	White	12	Part Time	Never Married	DNR	1	Non-Sinusoidal	DNR	Emory

*Participants 2 and 6 did not have any recorded non-opioid related mental disorders.

[a] MHD = Mental Health Disorders

[b] D/A= Drug and Alcohol Use

[c] DD = Depressive Disorders (All Cause)

[d] N/A = Not Available

[e] DNR = Data Not Recorded

[g] PD = Panic Disorder
